# We Are Not Alone: Examining the Impact of a Teen Diabetes Day Camp

**DOI:** 10.3390/bs15030370

**Published:** 2025-03-15

**Authors:** Heidi Blaylock, Eddie Hill, Carter Leuba, Christina Aguilar, Carla Cox

**Affiliations:** Department of Health, Physical Education, and Recreation, Weber State University, Ogden, UT 84408, USA; heidiblaylock@mail.weber.edu (H.B.); carterleuba@mail.weber.edu (C.L.); christinaaguilar@weber.edu (C.A.); carlacox1951@gmail.com (C.C.)

**Keywords:** type 1 diabetes, time in range, self-determination theory, medical specialty camps, positive youth development

## Abstract

Type 1 diabetes (T1D) is a chronic disease that influences all health aspects. The self-determination theory (SDT) suggests that three psychological needs of competence, autonomy, and relatedness are necessary for motivation to engage in healthy behaviors. Through medical specialty camps, these needs can be met by educating campers on how to manage T1D and realize they are not alone. The volunteer-based, five-day, inaugural tween–teen camp for youth with T1D was held on the campus of a university. The entire camp experience was engineered around the three basic needs described by the SDT. These needs were promoted by physical and educational activities and meeting friends. The Basic Psychological Needs and Frustration Scale was administered as a pre-, post-, and follow-up test. Two of the four measures were significant, the autonomy pretest (*M* = 3.93; *SD* = 0.75) to posttest (*M* = 4.49; *SD* = 0.56), with *t*(25) = −6.258; *p* ≤ 0.001) and relatedness pretest (*M* = 4.31; *SD* = 0.79) to posttest (*M* = 4.53; *SD* = 0.49), with *t*(25) = −2.168; *p* = 0.040; however, mean scores declined at the 3-month follow-up. Campers’ blood glucose levels were collected through an online platform that allowed staff to monitor blood glucose levels, with the week’s average being 150 mg/dL, and almost 71% remaining in the TIR. The results can be helpful for practitioners who want to use the SDT to structure and examine the effectiveness of medical specialty camps for youth with T1D.

## 1. Introduction

Type 1 diabetes (T1D) is a chronic disease that affects physical, mental, and social health. It not only affects the person with the disease but the entire family as well (Abdoli et al., 2020; Anarte et al., 2020; Bultas et al., 2016). The tedium of treatment regimens such as monitoring glucose levels along with feelings of isolation can decrease a person’s quality of life (Anarte et al., 2020). According to the Center for Disease Control and Prevention (CDC), there are 304,000 people under the age of 20 with T1D in the U.S. (Centers for Disease Control and Prevention, 2022) and no cure. The diagnoses worldwide are estimated to double among all ages by the year 2040 (Gregory et al., 2022).

To develop self-management of long-term health behaviors like diabetes, a person must be intrinsically motivated (Ryan & Deci, 2020). The self-determination theory (SDT) is based on three psychological needs of competence, autonomy, and relatedness. When these three needs are met, then the motivation for self-management of a chronic disease is internalized, thus resulting in healthier behavior (Lee et al., 2021). Medical specialty camps have demonstrated success at increasing camper well-being in all areas of health including intellectual, social, and mental (e.g., Allen et al., 2021; Bultas et al., 2016; Collins et al., 2021a; Hill et al., 2022). With the expected diagnoses to double by 2040, and no cure on the horizon, we need to be effective at providing healthy recreation education experiences for our youth. Therefore, the purpose of this study was to use the SDT to structure and examine the effectiveness of medical specialty camps for youth with T1D.

## 2. Literature Review

### 2.1. Psychological Effects of Type 1 Diabetes

Type 1 diabetes (T1D) is a chronic, lifelong condition that requires rigid management through daily glucose monitoring and dosing of insulin, either by injections or through a pump (Anarte et al., 2020; Hatzir et al., 2023). Multiple studies have shown that the constant need to manage T1D can lead to psychological and social problems potentially leading to poor health outcomes (Anarte et al., 2020; Kiriella et al., 2021; Majidi et al., 2020; Robinson et al., 2018). The daily tasks associated with T1D self-management have been shown to lead to anxiety, depression, diabetes distress, and diabetes burnout (Abdoli et al., 2020; Kiriella et al., 2021; Robinson et al., 2018). Majidi et al. (2020) reports that due to these psychological factors, youth and adolescents with T1D are at a higher risk of suicide.

Kiriella et al. (2021) defines diabetes distress as the “negative emotions experienced by people living with diabetes” (p. 1) and diabetes burnout as “barriers to adherence to diabetes treatment, characterized by feelings of mental, emotional, as well as physical exhaustion” (p. 2). Multiple studies have demonstrated this leads to difficulty with managing diabetes and disconnection from support systems and self. These feelings and lack of optimal diabetes self-care can lead to an increase in complications and poor outcomes (Abdoli et al., 2020; Kiriella et al., 2021; Robinson et al., 2018).

Robinson et al. (2018) expands on this concept and states that, along with diabetes distress and burnout, there are a myriad of mental health challenges that may arise (e.g., depression, anxiety, sleep, eating) in adolescents with T1D. “The presence of psychological symptoms and diabetes problems in children and adolescents with type 1 diabetes are often strongly affected by caregiver/family distress” (p. S133). The American Diabetes Association (2021) describes that foundations built on maintaining psychological well-being and positive health behaviors are fundamental in order to achieve T1D treatment goals and maximize quality of life.

### 2.2. Self-Determination Theory

The self-determination theory (SDT) is a motivation theory, which states that there needs to be satisfaction in three areas in order to influence behavioral changes. Ryan and Deci (2020) identified these areas as competence—“feeling of mastery”, autonomy—“sense of initiative and ownership in one’s actions”, and relatedness—“sense of belonging and connection” (p. 1). The motivation can come from within (intrinsic) or from outside (external). Collectively, when the three psychological needs are met, one begins to internalize the behavior.

Multiple studies have demonstrated that when youth with T1D are offered competence, autonomy, and relatedness, they are more likely to internalize behavior and are able to better self-manage the chronic condition (Allen et al., 2021; Arrington et al., 2023; Harvey et al., 2023). This leads to better glucose monitoring, taking of insulin, following a meal plan, participating in physical activity, and positive health outcomes. It is vital for youth to develop positive self-management and self-care of their diabetes (Hatzir et al., 2023; Lee et al., 2021; Stephens et al., 2021). Additionally, the SDT suggests providing autonomy-supportive environments to be most effective. These environments require three criteria: rational provision which focuses on sharing why, when difficult choices are needed, perspective taking, empathizing with individuals, and choice provision includes providing various options are needed to leave room for choice. This approach has been previously used with success among parents of youth with T1D (Barmoh et al., 2024). Diabetes camps are an authentic space to use this framework.

Diabetes camps are one place where competence, autonomy, and relatedness can be developed in youth with T1D through an autonomy-supportive environment. Some ways that camps for youth and adolescents with T1D can promote SDT include, but are not limited to, physical, creative, and educational activities, and close monitoring of glucose levels, carbohydrate intake, and insulin dosage (Kietaibl et al., 2024). By grounding diabetes camps in Positive Youth Development (PYD) and focus theory-based programming, evidenced-based practices can be created and scaled (Hill et al., 2022).

### 2.3. Positive Youth Development

Positive youth development (PYD) is widely used and accepted by recreation professionals as a framework for structuring programs to achieve positive outcomes (Shek et al., 2019). PYD is an approach that uses the concept of implementing positive environments and positive peer influences to foster well-being for the youth (Damon, 2004). Manning et al. (2018) states that “Peers may be more influential than diabetes educators, nurses, or doctors in helping youth manage the day-to-day difficulties of living with diabetes” (p. 252). Providing a medical specialty camp, using PYD for youth with T1D creates an environment for social support from peers in regard to diabetes management, which, in turn, helps improve the quality of life for youth that attend. This also may help improve the quality of diabetes self-management skills (Manning et al., 2018).

Along with PYD, Outcome-Focused Programming (OFP) can be used to identify theory-based outcomes of the program. “OFP is an approach utilized by recreation professionals to design and execute recreational experiences grounded in specific goals” (Hill et al., 2022, p. 162). Utilizing PYD and OFP, professionals can design an environment that benefits the participants’ well-being, as well as helping the participants leave with an increased knowledge of diabetes management skills and a sense of competence in their ability to perform these in the day-to-day activities of life (Hill et al., 2022; Weissber-Benchell & Rychlik, 2017).

### 2.4. Medical Specialty Camps

There are many benefits of summer medical specialty camps for youth and adolescents with T1D to include but are not limited to an increase in quality of life and self-esteem with a decrease in anxiety (Anarte et al., 2020). These camps help participants increase their diabetes self-care skills and gain education such as carbohydrate counting or coping with diabetes distress and burnout. They also provide an opportunity for participants to interact and build relationships with others who have been diagnosed with T1D (Collins et al., 2021b; Gagnon et al., 2019; Manning et al., 2018).

It is important for youth and adolescents with T1D to gain intrinsic positive motivation for the self-care and management of this life-long disease (Hatzir et al., 2023). The SDT is one model to foster this process (Lee et al., 2021; Ryan & Deci, 2020; Stephens et al., 2021). Medical specialty camps help aid youth and adolescents gain motivation through education, physical activity, and connection with others, ultimately leading to improved self-care; (Allen et al., 2021; Anarte et al., 2020; Bultas et al., 2016; Arrington et al., 2023; Kietaibl et al., 2024; Gagnon et al., 2019). Therefore, the purpose of this study was to use the SDT to structure and examine the effectiveness of medical specialty camps for youth with T1D. 

## 3. Methods

### 3.1. Program

Recreate, Educate, Advocate and Climb Higher (REACH) Weber is a year-around diabetes recreation program held on a college campus. The inaugural tween–teen camp for youth with T1D was held on the campus of a mid-west university. The camp ran for five days in August 2023, from 9 am to 4 pm each day. The activities for the camp included activities such as crafts, hiking, swimming, yoga, rock wall climbing, and educational workshops (e.g., pump training, stress management, etc.). During lunch time and with the snack, the nutrition information was provided to campers to promote autonomy and competence in calculating insulin dosage. The activities were chosen to encourage competence, autonomy, and relatedness among campers. The camp was facilitated by volunteers who consisted of members of volunteer service organizations, healthcare providers, university faculty and students, and community members. The program was funded through partnerships. These partnerships play a vital role in providing the opportunity for evidence-based research and making the program accessible for all participants.

### 3.2. Participants

Through a convenience sample, the participants were selected from existing diabetes camp areas along the Wasatch Front and consisted of 32 campers with T1D. The number of campers varied daily due to other summer activities. The mean age of the campers was 12.4 years. There were 16 females (52%), 15 males (48%), and one non-binary camper. There was one camper who was deaf for which accommodations were made. The median number of years since being diagnosed with T1D was two years.

### 3.3. Data Collection

After approval from the university Institutional Review Board, signed consent and assent forms were obtained. Data were collected using the Basic Psychological Needs and Frustration Scale (BPNSFS). The BPNSFS is a 24-item, 5-point Likert-type questionnaire that asks participants to self-report on areas of competence, autonomy, relatedness, as well as the level of frustration within each need. This new scale captures the satisfaction and frustration to support new findings regarding the absence of psychological needs; meaning satisfaction does not by definition imply no frustration (e.g., Ryan & Deci, 2017). The BPNSFS has been validated extensively among multiple cultures, but still needs further exploration among youth (Chen et al., 2015). Previous studies with adults demonstrated lower than expected reliability coefficients, under 0.70 (Šakan, 2022). The current study’s reliability coefficients were good: autonomy α = 0.87, relatedness α = 0.81, and competence α = 0.86. The BPNSFS was administered when campers arrived on the first day, the last day of camp, and three months later. The BPNSFS questionnaires were given in person and on paper. The statements ask campers about how they feel about their outlook on themselves. For example, outcome statements include “I often have doubts about whether I’m good at things”, “I feel free to choose which activities I do”, and “I feel close to and connected with the people who are important to me”.

Blood glucose level data were collected using an electronic medical record (EMR) called CampViews. CampViews provided an opportunity for the campers to connect their continuous glucose monitor (CGM) to the EMR, which allowed camp staff real-time tracking of campers’ blood glucose levels during the camp. Due to the glucose levels being in real-time, staff could make appropriate adjustments based on the reading of the camper’s blood glucose level. The campers were given snacks for low blood glucose levels or insulin correction doses for high blood glucose levels. This EMR allowed camp staff to better manage the camper’s T1D during the camp.

Time in range (TIR) is the percentage of the time that blood glucose levels fall within the target TIR of 70% or more. The TIR was calculated using an Excel formula, as this percentage is not available on CampViews (see Table 1). For the purposes of this research, researchers were only interested in the blood glucose levels and TIR for the hours during the camp. All information gathered from the questionnaires and TIR data were used to explore the use of the SDT to examine the effectiveness of a new diabetes camp for youth.

### 3.4. Qualitative Data

Open-ended questions were also added to the BPNSFS. These questions inquired about how much youth enjoyed the camp, how many friends were made, and favorite and least favorite activities. The final set of questions asked about new knowledge (regarding diabetes) learned while at camp. The additional data allowed youth to share other parts of their camp experience not asked from the BPNSFS. These rich data also would be used to help program future camp experiences for youth with T1D.

## 4. Results

### 4.1. Demographics and Qualitative Data

Thirty-two campers attended camp for the week. Twenty-six out of thirty-two campers (81% response rate) completed both the pre- and posttest. The majority of the campers were female (53%), white (91%), and the average age of the campers was 12.5 years. The average time diagnosed with T1D reported by campers was two years. On average, campers’ level of enjoyment was 9.56 (1–10 scale), they made 4.5 new friends, and their favorite activity was Gaga Ball. The most commonly reported new diabetes knowledge learned at camp was general knowledge of pumps and how to use them.

### 4.2. Paired Sample t-Test

A paired sample *t*-test was used through SPSS V28 to calculate composite scores for autonomy, relatedness, competence, and diabetes management (Chen et al., 2015). As seen in Table 2, two of the four variables (autonomy and relatedness) were significant. The composite scores for Autonomy (A) were as follows: pretest (*M* = 3.93, *SD* = 0.75) to posttest (*M* = 4.49, *SD* = 0.56), with *t*(25) = −6.258, *p* =< 0.001), effect size *r* = 1.2 (large). The composite scores for Relatedness (R) were as follows: pretest (*M* = 4.31, *SD* = 0.79) to posttest (*M* = 4.53, *SD* = 0.49), with *t*(25) = −2.168, *p* = 0.040), effect size *r* = 0.4 (small to medium). The composite scores for Competence (C) were as follows: pretest (*M* = 4.08, *SD* = 0.99) to posttest (*M* = 4.34, *SD* = 0.71), with *t*(25) = −2.016, *p* = 0.055), effect size *r* = 0.4 (small to medium). The composite scores for Diabetes Management (DM) were as follows: pretest (*M* = 4.19, *SD* = 0.74) to posttest (*M* = 4.24, *SD* = 0.63), with *t*(25) = −0.416, *p* = 0.681), effect size *r =* 0.08 (small).

### 4.3. Relationships Between TIR, Psychological Variables, and Sex

Paired samples *t*-test results showed no significant difference from Time 1 (Mon) and Time2 (Fri) on either glucose or TIR. There are two gender variables (sex, CmprGndr). The sex variable refers to analyses with the BPNSFS instrument, and the CmprGnder refers to analyses for the glucose/TIR variables. When you look at boys and girls and the BPNSFS subscales, there are no significant differences between boys and girls. There were no significant differences between boys and girls on the subscales (Autonomy, Relatedness, and Competence). No significant differences were found between boys and girls on the difference from T1 to T2 on each of the subscales. When you look at boys and girls and the glucose difference (T1 to T2) and the TIR difference (T1 and T2), there is still no significant difference. The only significant correlation between Autonomy, Relatedness, and Competence and either glucose level or time in range was a moderate to strong negative correlation (r = −0.627, *p*-value = 0.007) between Relatedness and TIR difference from T1 to T2. As Relatedness goes up, the TIR difference from Time 1 to Time 2 goes down.

### 4.4. Repeated Measures t-Test

The REACH Weber program is offered year-around; at three months post-camp, the BPNSFS was administered to the campers who attended the program in November (three months after summer camp). Eleven campers who attended the follow-up REACH session also attended the summer camp. Of those eleven, only one camper attended all three sessions post summer camp (September, October, and November monthly sessions). Of the other 10 campers, 50% attended two sessions, and the remaining 5 campers only attended one monthly session. A repeated measures test was conducted on these 11 campers from pre-, post-, and follow-up. Mean scores for all four outcome variables decreased.

### 4.5. Glucose Levels and Time in Range

Twenty-one out of thirty-two (66%) campers had their CGM connected with CampViews. Throughout the week, the campers maintained an overall average blood glucose level of 150 mg/dL, which falls within the target range of 70–180 mg/dL. The overall average TIR was 71%, which falls within the target range of 70% or more. Wednesday had the highest TIR at 78% (see Table 3). Activities on Wednesday included climbing, field games, and a water obstacle course.

## 5. Discussion and Implications for Practice

The purpose of this study was to use the SDT to structure and examine the effectiveness of medical specialty camps for youth with T1D. According to the CDC, there are around 304,000 people under the age of 20 with T1D in the U.S. (Centers for Disease Control and Prevention, 2022), and diagnoses worldwide are estimated to double by the year 2040. Due to the number of children being diagnosed with T1D increasing at such a fast rate, and no cure, there is cause for concern regarding the quality of life of the children with T1D and their families (Gregory et al., 2022). Medical specialty camps, like in this current study, could help alleviate some of the management challenges.

### 5.1. Theoretical Implications

The statistical significance of autonomy at posttest was promising. The camp experience provided lots of choice throughout the week (e.g., diabetes management, rock climbing, crafts, food, etc.). The campers were encouraged to participate in all planned activities but had choices when selecting activities. Autonomy-supportive language was also used whenever possible regarding diabetes management, as suggested by other research (Allen et al., 2021). For example, when discussing a camper’s blood sugar, camp staff would respond by asking what could/should be done to lower or increase, rather than telling campers what to do. Literature indicates that the use of autonomy-supportive environments has been successful in shifting behavior in a variety of settings such as sports, workplace, school, etc. (e.g., Ryan & Deci, 2020).

The statistical increase in relatedness was not surprising, given the nature of the camp experience. Youth with T1D are approximately 1/300; they are often the minority regarding their chronic condition when among peers. During such camps is the one time when they are in the majority. This space reinforces the concept of not being alone. Other research in the T1D space supports experiences like camps to help youth connect with others and become more resilient (Anarte et al., 2020; Williams et al., 2024). Campers did participate in all the activities but also took time away from participating to connect with their peers in unstructured opportunities. This positive social connection with peers increased the relatedness score. These findings are similar to other research on recreation programs for youth with T1D (Arrington et al., 2023; Harvey et al., 2023). Unlike competence and autonomy, which had to be intentionally programmed into the camp environment, relatedness was a direct result of bringing youth with T1D together in a positive environment.

The self-reported scores of competence and diabetes management were non-significant at posttest. Although campers identified learning new pump information, tried new activities, and shared knowledge of their own, more research is needed in the structure of promoted competence and diabetes management at camp. One reason scores did not indicate a significant increase might be due to dosage. Campers might need longer than a week-long camp to help realize what was learned, or a stronger focus on the structured learning might be required. Other diabetes recreation programs have demonstrated increased scores of competence, but were longer in duration (e.g., Allen et al., 2021). Future studies might want to adopt and implement learning modules from the American Diabetes Association as structured knowledge-based information. One note is the 6-item Diabetes Management measure, which is a new measure that needs a larger sample size to test the psychometric properties.

The no significant difference found between Monday and Friday regarding TIR is not surprising. For these types of behavioral changes, it may take months to show significant lifestyle changes as new behaviors are internalized. The lack of difference among males and females regarding the BPNSFS or TIR supports programming for all youth similarly. The unique significance of finding a negative correlation between relatedness and TIR difference from T1 to T2 needs to be further explored. As relatedness goes up (or connectedness with others), TIR difference from Time 1 to Time 2 goes down. This could be that the campers’ comfort level with camp and friends relaxed their vigilance. Further research is needed in this area.

The three-month follow-up lacked significance, but should be pursued. The decrease in sample size from the end of camp to the three-month follow-up could have impacted the statistics. Taking measures at 3, 6, 9, and 12 months needs to be continued to determine long-term impacts of the diabetes camp experience, especially for the diabetes programs that take place year-around (like the current study). These “touch points” of meeting monthly are extremely unique for diabetes recreation programs. There are only a few year-round programs like this in the U.S., and they are in high demand, but evidence-based practices are needed to statistically support their efforts (Allen et al., 2021; Barmoh et al., 2024; Hill et al., 2022).

### 5.2. Time in Range

The current study found that the average TIR for the week was 71%, with Wednesday being the highest TIR (78%), which aligns with the American Diabetes Association recommendations of remaining in range 70% of the time. The campers exerted a vigorous amount of physical activity (e.g., rock wall, field games, swimming, and water obstacle course) while participating in the activities on Wednesday. Eleven out of the twenty (55%) campers’ TIR data for Wednesday were greater than 80%, and two campers had a TIR at 100%. Measuring TIR has become the most effective tool, rather than HbA1c, which was historically used to determine effective management. While many camps collect TIR data, it is typically only used to treat blood sugar as needed (its purpose). However, very little literature exists on monitoring blood sugar for the duration of camp. This approach can add significant value to using EMR data and analyzing the camp experience to determine its efficacy.

### 5.3. Limitations and Future Research

Although the findings of this study were shown to be impactful and have positive implications for practice, there are limitations to the study. First, the campers did not stay overnight, as this was not a residential camp. Second, on some days, a few campers left early or came late due to other summertime activities such as sports. Third, not all campers completed both the pre- and posttests. The BPNSFS questionnaire was self-reported. Fourth, not all campers had their CGM connected to CampViews, so blood glucose level data were not collected from all campers.

There are a few recommendations for future studies. One, follow-up with the campers at various intervals of time after camp (e.g., six-months and 12-months) could be carried out to determine if they are continuing, at home, the knowledge (e.g., healthy choices, positive social connections) they learned at camp. The three-month follow-up failed to demonstrate significance; more time might have been needed. Researchers might also want to explore a more robust qualitative approach to data collection (e.g., Interpretative Phenomenological Analysis). Two, provide more deliberate learning and educational opportunities to help increase competence and diabetes knowledge gained by the campers. Three, provide greater opportunities at camp for a youth-lead/youth voice approach to diabetes education, physical activities, and other activities such as crafts.

### 5.4. Conclusions

This study used previous models and explored the use of the SDT to examine the effectiveness of a new diabetes camp for youth (e.g., Arrington et al., 2023). Autonomy and relatedness were both statistically significant and helped maintain Time in Range, suggesting that camp can be an effective way to increase motivation and improve diabetes management. This study adds to the body of research suggesting that diabetes camps can provide effective strategies for helping youth manage their diabetes and creating a Community of Practice (Hill et al., 2022; Weissber-Benchell & Rychlik, 2017). This study has implications for other medical specialty camps by demonstrating the use of the SDT and PYD to create an inviting environment for youth to thrive. The positive social connections that were built between campers, their peers, and volunteers during camp grew from having a safe space where participants felt safe, cared for, could be themselves, and where they knew that they were not alone.

The purpose of this study was to use the SDT to examine the effectiveness of medical specialty camps for youth and adolescents with T1D. With the rate of diabetes diagnoses in children rising at an alarming rate, and diagnoses expected to double worldwide in less than 15 years, there is concern for the quality of life for the children and their families. This study bridged theory and practice, creating a new space for youth with T1D to thrive and become resilient (e.g., Williams et al., 2024; Anarte et al., 2020). By building off the motivational SDT research, this study adds significant value to medical specialty camps, positive youth development, and health literature. The findings from this study demonstrate that medical specialty camps can have a positive effect on our youth with T1D, thus improving their quality of life.

## Figures and Tables

**Table 1:** Time in range formula.

Step 1	(determining if blood glucose level was between 70–180)	(=IF(AND(B9 > 70, B9 < 180), 1, 0)
Step 2	(determining if a data point was present at that point of time)	=IF(NOT(ISBLANK(B7)), 1, 0)
Step 3	(calculating the percentage of TIR)	=SUM()/SUM())

**Table 2:** Calculated composite scores.

	Pretest	Posttest	*t*(25)	*p*	*r*
Autonomy (A)	*M* = 3.93, *SD* = 0.75	*M* = 4.49, *SD* = 0.56	−6.258	<0.001	1.2 (large)
Relatedness (R)	*M* = 4.31, *SD* = 0.79	*M* = 4.53, *SD* = 0.49	−2.168	0.040	0.4 (small to medium)
Competence (C)	*M* = 4.08, *SD* = 0.99	*M* = 4.34, *SD* = 0.71	−2.016	0.055	0.4 (small to medium)
Diabetes Management (DM)	*M* = 4.19, *SD* = 0.74	*M* = 4.24, *SD* = 0.63	−0.416	0.681	0.08 (small)

**Table 3:** Percentages of time in range with activity.

	AVG. Blood Glucose	% Time in Range	Camp Activities
Monday	159	62%	Pool
Tuesday	153	73%	Hike
Wednesday	146	78%	Water obstacle course
Thursday	144	72%	Kickball
Friday	149	72%	Scavenger hunt

## Data Availability

The raw data supporting the conclusions of this article will be made available by the authors on request.
